# Simulation, Measurement, and Optimization of Sound Absorption in Nanofiber Membrane Composite with a Nonwoven Material

**DOI:** 10.3390/polym17070874

**Published:** 2025-03-25

**Authors:** Xiaofei Shao, Xiong Yan

**Affiliations:** Key Laboratory of Textile Science and Technology, Ministry of Education, College of Textiles, Donghua University, Shanghai 201620, China; shaoxiaofei@mail.dhu.edu.cn

**Keywords:** low-frequency, sound absorption mechanism, micro-perforated nanofiber membranes, nonwoven fiber felt, composites

## Abstract

To address the increasingly complex demands of noise control, this study investigated the integration of a micro-perforated nanofiber membrane (MPNM) with nonwoven fiber felt (NFF), exploiting their synergistic effects to achieve efficient low-frequency broadband sound absorption. Through theoretical analysis, numerical simulations, and experimental validation, the relationship between the sound absorption performance of the composite structure and factors such as the lamination sequence, bonding area, perforation parameters, thickness of the MPNM, and thickness of the NFF were elucidated. These findings provided new insights for the design of high-performance, tunable, sound-absorbing materials. The results demonstrated that the MPNM-NFF effectively combined two distinct sound absorption mechanisms, thereby expanding the effective absorption bandwidth, with particularly enhanced low-frequency sound absorption. Moreover, through algorithmic optimization of the structural parameters, targeted absorption of noise across different frequency bands was achieved, with the optimal average sound absorption coefficients reaching 0.70 in the low-frequency range, 0.91 in the mid-frequency range, and 0.82 in the full-frequency range. This research offered both theoretical foundations and practical guidance for the development of composite materials with high efficiency and broadband sound absorption characteristics, paving the way for innovative applications in noise control materials.

## 1. Introduction

With the development of modern industrial civilization, noise pollution, as an invisible form of environmental pollution, permeates various aspects of urban life and increasingly threatens human health and quality of life [1,2,3]. Among the various measures to address noise pollution, sound absorption and noise reduction technologies have gained significant attention due to their direct and effective nature. Textiles, as important materials in the field of sound absorption and noise reduction, possess inherent lightweight and porous structures that provide excellent sound-absorbing properties [4,5,6]. Polyester (PET) fiber felt, a representative nonwoven material, combines lightweight, porous, and flexible properties, making it especially effective for sound absorption and noise reduction applications. Furthermore, PET fiber felt possesses excellent mechanical properties and chemical stability, enabling the formation of robust composite structures with nanofiber membranes. This ensures the durability and reliability of the material in practical applications. Additionally, its low cost and ease of processing make it an ideal candidate for large-scale production and application, positioning it as one of the most widely used sound-absorbing materials in the market today [7]. However, due to the inherent mechanisms of porous sound absorption, nonwoven fabrics made from coarse fibers typically exhibit excellent sound absorption performance in the high-frequency range. Within the low and medium frequency range of 100–2500 Hz, sound waves with longer wavelengths and slower attenuation require larger structural dimensions for effective absorption, which limits their performance in practical applications [8].

With advancements in nanotechnology, nanofiber membranes have gradually become key materials for efficient noise suppression in various acoustic environments. Their unique structural characteristics, including an ultra-high specific surface area and porosity, endow them with exceptional sound-absorbing properties, effectively enhancing low-frequency sound absorption while maintaining a lightweight design [9]. However, the electrospinning process faces low efficiency [10], which limits its application in sound absorption and noise reduction. Polyvinyl butyral (PVB) has been shown to increase the spinning speed while maintaining process stability, achieving up to 30 times the spinning rate of other conventional electrospinning materials [11] and significantly improving the efficiency of sound-absorbing material production. Notably, PVB possesses a range of outstanding properties, including a low cost, non-toxicity, light resistance, and other properties that make it ideal for sound absorption applications.

Micro-perforated structures, which are widely used for low-frequency noise absorption, have been extensively studied due to their ability to absorb sound through particle resonance within micro-holes and viscous dissipation effects [12]. To further enhance sound absorption, micro-perforated nanofiber membranes (MPNMs) have been developed, combining electrospinning technology with micro-perforation processes. This dual-porosity structure, which integrates micro-holes and nanofibers, offers superior sound absorption performance compared to traditional micro-perforated panels [13]. However, MPNMs still face challenges in terms of broadband efficiency and lightweight design. Specifically, their sound absorption performance typically exhibits a single resonant peak, leading to a narrow effective absorption bandwidth. Expanding this bandwidth remains a key area for improving overall performance.

As noise control requirements become increasingly complex, single materials are insufficient, prompting the development of composite materials with diverse structural characteristics [14]. The combination of different materials can produce synergistic effects, improving sound absorption efficiency and broadening the absorption bandwidth, particularly in low- and mid-frequency ranges [15]. Numerous studies have demonstrated that coating nanofiber membranes onto fabrics [16], foams [17], and micro-perforated panels [18] can improve sound absorption performance. However, despite advancements in composite structures, designing and optimizing material structures to meet specific application needs remains a major challenge. The performance of composite materials depends not only on the properties of their constituents but also on their structural parameters. Thus, further exploration is needed to optimize these parameters for superior broadband efficiency, high-performance absorption, and lightweight design.

This study combined the resonant sound absorption mechanism of an MPNM with the porous absorption mechanism of nonwoven fiber felt (NFF), developing a fiber-based composite sound-absorbing structure with multiple absorption mechanisms. This composite structure enabled the efficient absorption of noise across a wide frequency range, utilizing a lightweight, thin, flexible fiber framework. MPNMs were fabricated using electrospinning and micro-perforation processes. These membranes were then combined with a low-cost, high-performance PET needle-punched NFF to form a composite structure. To fully leverage the advantages of both materials and achieve superior acoustic performance, this study systematically investigated the effects of the lamination sequence, bonding area, perforation parameters, nanofiber membrane thickness, and backing fabric thickness on sound absorption performance. The study emphasized the acoustic mechanism analysis and structural optimization of the composite, revealing the relationship between structural parameters and sound absorption performance through theoretical derivation and numerical simulations, providing a scientific basis for the further application of composite materials.

## 2. Materials and Methods

### 2.1. Experimental Materials and Sample Preparation

**Figure 1:**
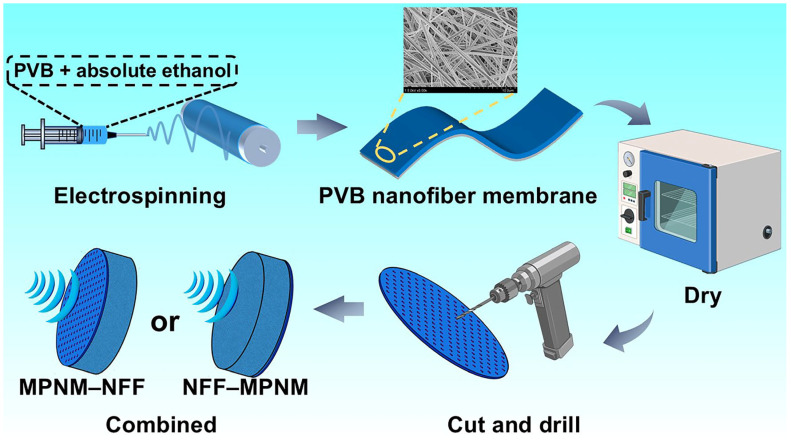
Preparation of the micro-perforated nanofiber membrane composite with nonwoven fiber felt.

PVB and anhydrous ethanol were both purchased from Shanghai Titan Technology Co., Ltd. (Shanghai, China). The PVB powder was dissolved in anhydrous ethanol, and the solution was stirred at room temperature using a magnetic stirrer to form a homogeneous solution with a concentration of 8 wt%. PVB nanofiber membranes were prepared by electrospinning, with the spinning voltage set at 20 kV, the solution feeding rate set at 4 mL/h, and the collection distance at 15 cm. The nanofiber membranes obtained were dried in an oven at 60 °C for 24 h. Subsequently, MPNMs with varying perforation parameters were fabricated using a mechanical drilling method. The PET nonwoven fabric, purchased from Changshu Jingshuang Decorative Materials Co., Ltd. (Changshu, China), has a fiber diameter of approximately 25 μm. The MPNMs were freely laid on one side of the NFF under no tension, ensuring a tight bond between the two materials to form a micro-perforated nanofiber membrane composite nonwoven fiber felt sound-absorbing structure. The overall preparation process of the composite is illustrated in Figure 1.

### 2.2. Characterization of the Micro-Perforated Nanofiber Membrane Composite with Nonwoven Fiber Felt

The air permeability of the composite sound-absorbing structure was tested according to the ASTM D737-2018 standard [19] using the YG461E digital fabric permeability tester manufactured by Wenzhou Baien Instrument Co., Ltd. (Wenzhou, China). The tests were conducted under standard environmental conditions, with a room temperature of 21 ± 1 °C and a relative humidity of 65 ± 2%. The thickness of each sample was measured according to the GB 3820-1997 standard [20] using a YG141D-II fabric thickness tester manufactured by Ningbo Dahe Instrument Co., Ltd. (Ningbo, China). The thickness tests were also conducted under standard environmental conditions. For each sample discussed in this chapter, three valid measurements were taken under the same conditions, and the average value was used for analysis.

### 2.3. Sound Absorption Performance Test

The sound absorption coefficients (SACs) of the composite structure in the 100–2500 Hz range were tested using an SW 260 two-microphone impedance tube manufactured by BSWA Technology Co., Ltd. (Bejing, China). Based on its reliability, compatibility with available equipment, and widespread use in similar studies, all acoustic parameters presented, calculated, and tested in this study were determined according to the ISO 10534-2 standard [21] (Acoustics—Determination of sound absorption coefficients and impedances in impedance tubes: Transfer function method). The formula for calculating the average sound absorption coefficient is as follows:

(1) $$\bar{\alpha }=\frac{\int_{F_{1}}^{F_{2}}\alpha (f)df}{F_{2}-F_{1}}$$

where *F*_1_ and *F*_2_ represent the start frequency and cutoff frequency, respectively.

## 3. Results and Discussion

### 3.1. Effect of the Lamination Sequence on the Sound Absorption Performance

**Figure 2:**
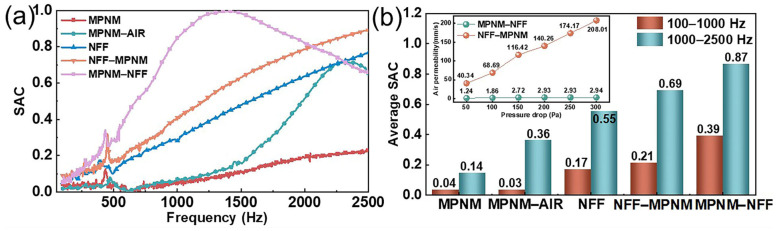
Effect of the lamination sequence on the sound absorption performance of the composite structures: (**a**) SAC curves and (**b**) average SAC (the inset in the upper left corner shows the air permeability of the corresponding samples).

Both the intrinsic properties of the materials and the lamination sequence have significant impacts on the sound absorption performance of composite structures. Considering the property differences between MPNMs and NFF, the sound-absorbing structure formed by their combination provided two distinct sound propagation paths, as shown in Figure 1. One path follows the direction of the NFF, labeled NFF-MPNM, and the other follows the direction of the MPNM, labeled MPNM-NFF. To investigate the sound absorption performance and mechanisms of the composite structure under these two propagation paths, the normal incidence sound absorption coefficient for different wavefront orientations was tested, with the results shown in Figure 2. The structural parameters of the different samples are provided in Table 1.

Compared to single-phase sound-absorbing materials, composite structures exhibit superior broad-band acoustic absorption characteristics by synergistically constructing a gradient acoustic impedance through the heterogeneous porosity of the MPNM and NFF. As illustrated in Figure 2, when the NFF served as the incident surface for sound waves (propagating in the NFF-MPNM direction), the composite system demonstrated typical porous sound absorption behavior, with the SAC increasing monotonically with frequency. Compared to the NFF alone, the addition of a thin MPNM significantly enhanced its sound absorption performance, particularly in the mid-frequency range. This optimization resulted in a 25.5% increase in the average SAC with only a 5% increase in thickness. The underlying mechanism involved dual dissipation pathways: the NFF layer at the front, with its low acoustic impedance, achieved excellent impedance matching with air, effectively reducing interface reflection losses. Meanwhile, the MPNM at the rear caused multiple sound reflections, extending the effective propagation path within the composite system. Such multiple reflections enhanced the viscous and thermal losses within the fabric, effectively dissipating sound energy. Since the composite structure still exhibited pronounced porous sound absorption characteristics, with the SAC increasing with frequency, the improvement in sound absorption performance was highly dependent on the thickness. This suggested that the enhancement of sound absorption came at the cost of occupying more space, and thus could not achieve broad-band noise absorption in limited spaces.

When the MPNM served as the incident surface, with sound waves propagating along the MPNM-NFF direction, the SAC curves of the composite structure exhibited a distinct trend compared to that of the NFF. Specifically, the SAC initially increased and then decreased with frequency, displaying a pronounced resonance absorption peak. This behavior indicated that the designed sound-absorbing structure overcame the limitations associated with the use of each material independently. The composite structure reached a peak SAC of 1.0 at 1402 Hz and maintained values above 0.9 within the frequency range of 1082 to 1756 Hz. The low-frequency average SAC was 0.39, while the mid-frequency average SAC was 0.87. Additionally, both the effective sound absorption bandwidth (α > 0.2) and the half-power absorption bandwidth (α > 0.5) were broadened to 2134 Hz and 1828 Hz, respectively. These improvements were primarily attributed to the loose structure of the NFF, which, when placed behind the MPNM, acted as a physical cavity backing, inducing micro-perforated resonance absorption in the composite structure. Furthermore, a comparison between the absorption performances of MPNM-AIR and MPNM-NFF revealed that, although both structures had an identical thickness of 10.5 mm, the composite structure filled with the NFF exhibited superior sound absorption performance compared to the simple air cavity. Specifically, MPNM-AIR lacked sound absorption in the low-frequency range and exhibited a mid-frequency average SAC of only 0.36. This underscored the superior sound energy dissipation capability of the micro-sized holes formed by the entangled fibers. In micro-perforated structures, filling the cavity with the NFF altered the internal medium, thereby modifying the acoustic impedance of the composite structure, as described by the following relationships [22]:

(2) $$C_{0}=\frac{V_{0}}{\rho_{0}c_{0}^{2}}$$

(3) $$\frac{C_{1}}{C_{0}}=\frac{\rho_{0}c_{0}^{2}}{\rho c^{2}}$$

(4) $$C_{1}=\beta C_{0}=\beta \frac{V_{0}}{\rho_{0}c_{0}^{2}}$$

where *C*_0_ is the acoustical compliance when the medium is air, *C*_1_ is the acoustical compliance when the medium is the NFF, *ρ*_0_ and *c*_0_ are the density and sound speed in the air, *ρ* and *c* are the density and sound speed when the NFF is the medium, *V*_0_ is the volume of the back cavity, and *β* is the heat-loss factor, for which the value is generally between 1 and 1.4. From Equations (2)–(4), we can see that when the back cavity was replaced by the NFF, the acoustical compliance of the microperforated structure increased by a factor of *β* accordingly. For composite structures, the relationship between acoustical compliance and resonant frequency can be obtained from the following equation [23]:

(5) $$f_{0}=\frac{1}{2\pi }\sqrt{\frac{1}{M_{1}C_{1}}}$$

where *f*_0_ is the resonant frequency and *M*_1_ is the acoustic mass of the composite structure. As indicated by Equation (5), the resonance frequency of the micro-perforated structure is inversely proportional to the acoustic compliance, implying that filling the cavity with fibers leads to a reduction in the resonance frequency. Additionally, the introduction of a porous sound-absorbing material into the cavity altered the radiation characteristics of the micro-holes, generating radiation impedance. This resulted in an effective increase in the hole length, further lowering the resonance frequency [24]. The combined effects of these two factors contributed to the enhanced sound absorption performance of the composite structure.

Finally, numerical comparisons demonstrated that the SAC curves exhibited significant differences when sound waves were incident from different surfaces of the composite structure. Compared to the NFF-MPNM structure, the MPNM-NFF structure showed superior sound absorption performance in the mid-to-low frequency range, particularly with an 83.5% difference in the average low-frequency SAC. However, its performance diminished in the high-frequency range beyond 2050 Hz. This discrepancy was attributed to the higher flow resistance of the MPNM. For the low-frequency range before the resonance peak, the absorption of low-frequency sound waves was positively correlated with the material’s flow resistance. Materials with higher flow resistance at the incident surface contributed to enhanced low-frequency sound absorption. In contrast, for the mid-to-high frequency range beyond the resonance peak, high-frequency sound waves experienced more reflection during penetration and failed to effectively enter the structure for energy dissipation. Additionally, the loose structure of the NFF did not effectively retain and dissipate sound energy, thereby impairing the sound absorption performance at higher frequencies. Furthermore, related studies have shown that the acoustic performance of fiber materials is closely linked to their air permeability, with reduced permeability contributing to better sound absorption [25]. As illustrated in the inset of Figure 2b, the air permeability of the NFF-MPNM structure was significantly higher than that of the MPNM-NFF structure, indicating that airflow was more easily transmitted through the composite material. For the NFF-MPNM structure, the acoustic wave penetrating the nonwoven on the incident side was reflected by the nanofiber membrane on the back side, but the loose nature of the nonwoven structure could not consume the acoustic energy efficiently, resulting in lower acoustic energy consumption. On the other hand, for the MPNM-NFF structure, sound waves penetrating through the MPNM and NFF reached the bottom layer, where they were reflected by the rigid back wall. The nanofiber membrane at the top layer effectively prevented sound waves from reflecting outward, allowing a portion of the sound to re-enter the structure. This cyclic motion of sound waves facilitated the effective dissipation of sound energy, thereby enhancing the material’s sound absorption performance.

### 3.2. Effect of the Bonding Area on the Sound Absorption Performance

**Figure 3:**
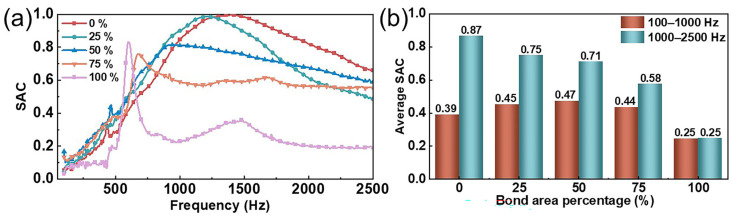
Effect of the bonding area on the sound absorption performance of composite structures: (**a**) SAC curves and (**b**) average SAC.

The bonding area between different material layers determines the impedance matching during sound wave transmission and the scattering of sound waves between the layers. An appropriate bonding area can not only enhance the adhesive strength of the material but also improve the sound absorption performance of the composite structure. Therefore, for the acoustic performance of multi-layer composite structures, the rational selection and design of bonding methods between the material layers is crucial. To investigate this effect, we fabricated MPNM-NFF composite structures with varying bonding areas, in which the perforation diameter was 0.8 mm, the perforation rate was 1%, the thickness of the MPNM was 0.5 mm and the thickness of the NFF was 10 mm. A polyamide hot-melt adhesive mesh was employed as an intermediary to facilitate the interface bonding between the MPNM and NFF. In the experimental procedure, the adhesive mesh was fabricated into annular structures of varying diameters, with the outer diameter consistently fixed at 40 mm. The adhesive area was precisely controlled by adjusting the inner diameter. Following precise alignment, the annular structure was positioned at the material interface and subjected to thermal bonding under heat and pressure using a glass plate at 120 °C. By finely tuning the geometric parameters of the annular structure, the adhesive area could be accurately and systematically regulated. The resulting sound absorption performance of the composite structures with varying bonding areas is depicted in Figure 3.

As shown in Figure 3, the bonding area between the MPNM and the NFF had a significant impact on the sound absorption performance of the composite structure. When the MPNM was simply laid on the NFF without any tension, the composite structure achieved a peak resonance absorption at 1402 Hz with a value of one, and the average SAC in the mid-frequency range reached 0.87. When the bonding area was 25%, the resonance frequency shifted to 1218 Hz, with nearly 100% noise absorption at the resonance frequency. As the SAC curve shifted towards lower frequencies, the average SAC in the low-frequency range increased from 0.39 to 0.45, and the effective sound absorption bandwidth expanded from 2132 Hz to 2166 Hz. This improvement was attributed to the viscoelastic properties of the adhesive, which reduced the free vibrations of the structure and lowered the resonance frequency. Furthermore, the adhesive did more than just bond the material layers together; it also altered the dynamic characteristics between the layers, providing additional rigidity and damping effects, which affected the vibration characteristics of the entire composite structure. As the bonding area increased, the resonance frequency shifted further towards lower frequencies, accompanied by a sharp decrease in the SAC. The absorption peak at the resonance frequency became smaller and sharper. When the bonding area reached 100%, the composite structure exhibited an absorption peak of 0.83 at 600 Hz, but the peak was very sharp. The sound absorption performance deteriorated outside the resonance frequency, with the half-power absorption bandwidth narrowing to only 100 Hz and the average SAC in the full-frequency range dropping to 0.25, significantly reducing the overall sound absorption performance. This phenomenon was fundamentally attributed to the interlayer dynamic constraint effects induced by excessive bonding. The theoretical analysis indicated that when the interface adhesive area exceeded a critical threshold, overly strong interlayer coupling significantly suppressed the relative slip motion between the MPNM and NFF. Moreover, the continuous extension of the adhesive interface eliminated the interlayer air gap structure, obstructing the sound wave–porous medium interaction path originally facilitated by Helmholtz resonance, which substantially diminished the viscous dissipation efficiency [26]. The experimental data further revealed that maintaining a moderate degree of interlayer mobility could synergistically activate multiple acoustic energy dissipation mechanisms. On one hand, periodic shear motion at the fiber–air interface enhanced thermoelastic losses. On the other hand, reciprocating friction at the contact surfaces improved the energy conversion efficiency.

In summary, the varying bonding areas between the MPNM and the NFF affected the transmission and dissipation of sound waves within the fibers. In practical applications, ensuring the structural stability and durability of the acoustic material is necessary to avoid delamination or peeling failure during the use process, to ensure long-term stable noise reduction performance, but also to take into account the loss of the acoustic performance of the composite structure. Therefore, considering both acoustic performance and structural stability, the composite structure exhibited optimal overall performance when the bonding area was 25%. This ratio ensured structural stability while maintaining excellent sound absorption efficiency.

### 3.3. Effects of Micro-Perforation Parameters on the Sound Absorption Performance

**Figure 4:**
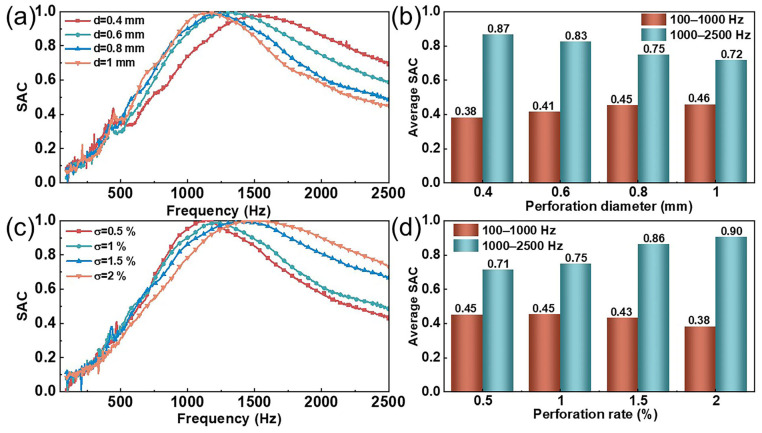
Effects of the perforation parameters on the sound absorption performance of composite structures: (**a**,**b**) perforation diameter and (**c**,**d**) perforation rate.

For micro-perforated structures, the perforation size and distribution determine the acoustic wave propagation characteristics of the material, which influence the reflection, attenuation, and energy conversion of sound waves. Therefore, to investigate the impact of perforation parameters on the sound absorption performance of composite structures, we fabricated MPNM-NFF composite materials with different perforation diameters and perforation rates. The thickness of the MPNM was 0.5 mm, and the thickness of the NFF was 10 mm, with a bonding area of 25% between the two layers. The sound absorption performance of the composite structures with different perforation parameters in the range of 100–2500 Hz is shown in Figure 4.

As shown in Figure 4a,b, when the perforation rate was 1% and the other structural parameters remained unchanged, within the perforation diameter range of 0.4 mm to 1 mm, an increase in diameter caused the resonance frequency of the composite structure to gradually decrease from 1500 Hz to 1130 Hz. This shift led to an increase in the low-frequency average SAC from 0.38 to 0.46, while the mid-frequency average SAC decreased from 0.87 to 0.72. This phenomenon could be attributed to the coupling effect between the perforation diameter and acoustic impedance. As the perforation diameter increased from 0.4 mm to 1 mm, the cross-sectional area of each perforation expanded by a factor of 6.25, significantly increasing the effective acoustic mass of the air column. Simultaneously, under the constraint of a fixed perforation rate, an increase in the perforation diameter led to a reduction in the number of micro-holes, which was inversely proportional to the square of the diameter, thereby increasing the equivalent acoustic compliance of each micro-hole. According to calculation Formula (4), increases in both the acoustic mass and compliance resulted in a decrease in resonance frequency. Notably, while expanding the perforation diameter enhanced low-frequency sound absorption, it weakened the impedance-matching effect, leading to a sharpening of the absorption peak and a narrowing of the absorption bandwidth. This provided important trade-off considerations for optimizing the parameters of the micro-perforated structure.

As shown in Figure 4c,d, when the perforation diameter was 0.8 mm and the other structural parameters remained unchanged, increasing the perforation rate from 0.5% to 2% caused the resonance frequency of the composite structure to gradually shift from 1132 Hz to 1516 Hz. Correspondingly, the low-frequency average SAC decreased from 0.45 to 0.38, while the average SAC in the mid-frequency range increased from 0.71 to 0.9. This was due to the fact that, with the perforation diameter held constant, increasing the perforation rate implied an increase in the number of holes, which reduced the coupled acoustic compliance of each hole. According to Equations (2)–(5), the reduction in the volume behind the micro-holes led to a decrease in acoustic compliance. Since the resonance frequency of the micro-perforated structure was inversely proportional to its acoustic compliance, increasing the perforation rate resulted in an increase in the resonance frequency. Notably, while an increase in perforation ratio expanded the acoustic impedance matching range in the mid-to-high frequency range, it simultaneously exacerbated the impedance mismatch effect in the low-frequency range. This caused the absorption peak to shift to higher frequencies and led to a divergence in the sound absorption characteristics across the frequency domain.

In conclusion, the sound absorption performance of MPNM-NFF, as macroscopic micro-perforated structures, was influenced by the perforation parameters. Within the range of values considered in this study, composite structures with small perforation diameters and high perforation rates exhibited good sound absorption performance in the mid-to-high frequency range, while composite structures with large perforation diameters and low perforation rates performed better in the low-frequency range. Therefore, when designing and applying micro-perforated panels, comprehensively considering the noise frequency range and the manufacturing difficulty is essential to achieve the optimal sound absorption performance.

### 3.4. Effect of the Thickness of the MPNM on the Sound Absorption Performance

**Figure 5:**
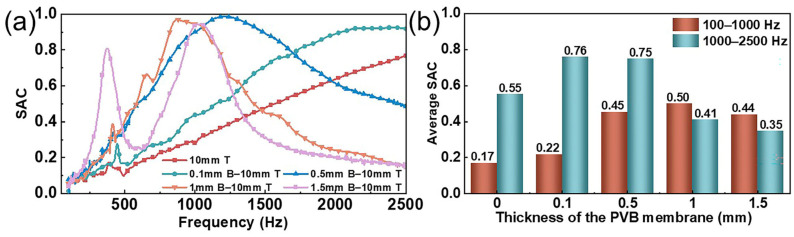
Effect of the thickness of the MPNM on the sound absorption performance of composite structures: (**a**) SAC curves and (**b**) average SAC.

The sound absorption performance of textile materials is closely related to their thickness. To investigate the effect of the thickness of the MPNM on the sound absorption performance of the composite structure, we fabricated MPNM-NFF composite materials with varying nanofiber membrane thicknesses. The perforation diameter was uniformly set to 0.8 mm, the perforation rate was 1%, the thickness of the NFF was 10 mm, and the bonding area between the two layers was 25%. The changes in the sound absorption performance of the composite structure as the thickness of the MPNM increased from 0 mm to 1.5 mm are shown in Figure 5.

As shown in Figure 5, compared to the NFF alone, the incorporation of a 0.1 mm thick MPNM in front of it significantly improved the sound absorption performance. The low-frequency average SAC increased to 0.22, and the average SAC in the mid-frequency range reached 0.76, representing increases of 29% and 38%, respectively. According to Figure 5a, when the thickness of the NFF remained constant, increasing the thickness of the MPNM led to a SAC for the composite structure that displayed a distinct trend compared to the NFF alone. Specifically, the SAC initially increased and then decreased as the noise frequency rose, resulting in a pronounced resonance peak. This indicated that the constructed sound absorption structure overcame the limitations of using each material individually, with the low-frequency average SAC rising to 0.45. As the thickness of the MPNM continued to increase, the SAC curve shifted further toward the low-frequency range, and the sound absorption bandwidth gradually narrowed. When the thickness of the MPNM reached 1.5 mm, the composite structure showed a peak SAC of 0.81 at 378 Hz, which resulted in an average SAC of 0.38 in the 100–500 Hz ultra-low frequency range, a 251% increase compared to the single nonwoven material. This was primarily due to the increase in the MPNM thickness, which extended the air movement path within the pores and increased its inertia, thereby increasing the acoustical mass of the composite structure. According to the concept of acoustic mass, the larger the equivalent mass of the vibrating system, the lower the system’s natural frequency, and thus the resonance frequency decreased accordingly. However, when the MPNM thickness became too large, the air friction loss within the holes decreased, the damping effect weakened, and the sound absorption bandwidth narrowed. Additionally, the increased thickness also meant greater resistance to sound wave penetration, causing more high-frequency waves to reflect during penetration and preventing effective absorption within the structure. As a result, the sound absorption performance of the composite structure in the mid-to-high frequency range was compromised.

### 3.5. Effect of the Thickness of the NFF on the Sound Absorption Performance

**Figure 6:**
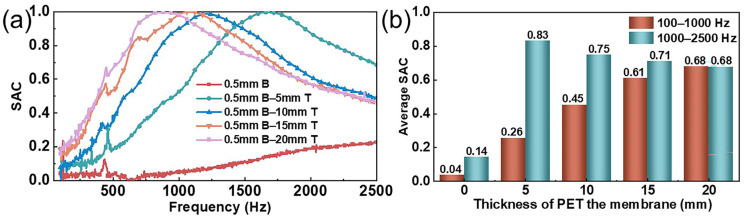
Effect of the thickness of NFF on the sound absorption performance of composite structures: (**a**) SAC curves and (**b**) average SAC.

To investigate the effect of the thickness of the NFF on the sound absorption performance of the composite structure, a one-factor experimental method was employed. Under the conditions of a perforation diameter of 0.8 mm, a perforation rate of 1%, a bonding area of 25%, and a MPNM thickness of 0.5 mm, the changes in the sound absorption performance of the composite structure were studied as the thickness of the NFF ranged from 0 mm to 1.5 mm. The results are shown in Figure 6.

As shown in Figure 6, the thickness of the NFF had a significant impact on the sound absorption performance of the composite structure. According to Figure 6a, when the thickness of the MPNM remained constant, increasing the thickness of the NFF caused the SAC curve of the composite structure to shift significantly toward lower frequencies. As the thickness of the NFF increased from 5 mm to 20 mm, the resonance frequency of the composite structure shifted from 1670 Hz to 880 Hz. Notably, the composite structure achieved 100% noise absorption near the resonance frequency, with the absorption peak remaining largely unchanged despite the reduction in the resonance frequency. This was because the NPNM-NFF belonged to a resonant sound absorption structure at the macroscopic level. The thicker the NFF, the larger the volume of each Helmholtz resonator, and as indicated by Equations (2)–(5), the acoustic compliance increased accordingly. Since the acoustic compliance is inversely proportional to the resonance frequency of the micro-perforated structure, the thicker the NFF, the lower the resonance frequency, thus shifting toward the low-frequency range. At the same time, since the relative acoustic impedance of the MPNM did not change, the peak absorption value remained essentially unchanged. Furthermore, as shown in Figure 6b, as the thickness of the NFF increased, both the effective sound absorption bandwidth and the half-power absorption bandwidth were broadened. When the thickness of the NFF reached 20 mm, the composite structure maintained a SAC above 0.9 in the frequency range of 668–1168 Hz. The low-frequency average SAC reached 0.68, and the effective sound absorption bandwidth and the half-power absorption bandwidth expanded to 2378 Hz and 1964 Hz, respectively, covering 99.1% and 81.8% of the full frequency range. The increase in the NFF thickness restructured the radiative impedance characteristics of the micro-perforated structure, significantly improving the radiated acoustic impedance and acoustic mass at the perforation ends, modifying the equivalent neck length, and lowering the resonance frequency, thereby expanding the impedance matching range [24]. Additionally, the increase in the thickness of the NFF meant an increase in flow resistance, which resulted in an increased acoustic impedance of the composite structure. This facilitated the absorption of mid-to-high frequency sound energy. Therefore, after the resonance frequency, the sound absorption coefficient did not drop below 0.2, thus broadening the sound absorption bandwidth and demonstrating the advantages of the composite structure.

In summary, the composite structure of the MPNM-NFF was able to fully leverage the advantages of both materials, resulting in enhanced sound absorption performance. By adjusting the lamination sequence, bonding area, perforation parameters, thickness of the MPNM, and thickness of the NFF, different sound absorption effects could be achieved. This composite structure demonstrated the ability to achieve lightweight, broadband noise reduction, making it of significant research importance in noise control and acoustic design.

## 4. Model Construction of the MPNM-NFF

In order to deeply analyze the physical mechanism of acoustic wave absorption and understand the influence of structural parameters on the acoustic performance of composite structures, the establishment of theoretical models is particularly important. In this study, the Transfer Matrix Method was employed as the analytical tool, where the entire system’s computational process was decomposed into calculations of multiple lower-order units. These units’ transfer matrices were then multiplied to obtain the overall acoustic characteristics of the system. When calculating the SAC of the MPNM-NFF in the 100–2500 Hz range, the sound impedances of the MPNM and the NFF needed to be computed separately, and the composite material’s sound impedance was obtained through transfer matrix operations. The establishment and analysis of the theoretical model provided not only a direction for experimental design but also a powerful tool for analyzing the experimental results. By combining theory and experiments, a more comprehensive understanding of the composite material’s acoustic performance could be achieved, providing effective solutions for noise control.

### 4.1. Transfer Matrix of the MPNM

The transfer matrix of the MPNM in the composite structure is

(6) $${\left[T\right]}_{PVB}=\left[\begin{array}{cc} 1 & Z_{PVB}\\ 0 & 1 \end{array}\right]$$

where *Z_PVB_* represents the sound impedance of the MPNM. For the calculation of the acoustic impedance of the MPNM, we need to analyze it for both macroscopic and microscopic aspects. On a macroscopic level, the MPNM was classified as a micro-perforated structure, and thus, the classic Maa model for calculating the SAC of micro-perforated materials [27] served as the foundation for model improvements. In the process of calculation, taking into account that the mechanical perforation method will lead the edge of the microporous structure to become not smooth and that there is a part of viscous loss is necessary. Therefore, the acoustic impedance calculation equation needs to be corrected to accurately reflect the complex acoustic properties of the microporous holes and the energy loss in the acoustic wave propagation process. Here, we refer to the expression of the end correction coefficient based on the geometry of microporous edges proposed by Temiz et al. [28] to correct the acoustic impedance calculation formula proposed by Maa. The acoustic resistance *r*_1_ and acoustic impedance *m*_1_ of the MPNM after correction are

(7) $$r_{1}=\frac{32\rho \mu t}{p\rho_{0}c_{0}d^{2}}(\sqrt{1+\frac{k_{m}^{2}}{32}}+\beta \frac{\sqrt{2}}{16}k_{m}\frac{d^{2}}{t})$$

(8) $$m_{1}=\frac{t}{pc_{0}}[1+{\left(3^{2}+\frac{k_{m}^{2}}{2}\right)}^{-\frac{1}{2}}+\frac{\delta }{2}\frac{d}{t}]$$

(9) $$\beta =5.08k_{m}^{-1.45}+1.7-\frac{0.002}{t^{*}}$$

(10) $$\delta =\delta s-\delta_{H}$$

(11) $$\delta_{S}=0.97\exp(-0.2k_{m})+1.54-\frac{0.003}{t^{*}}$$

(12) $$\delta_{H}=0.85\sigma -2.4\sqrt{\sigma }+1.54$$

(13) $$t^{*}=\frac{t}{d}$$

(14) $$z_{m}=r_{1}+j\omega m_{1}$$

where *η* is the viscous coefficient of air motion, *μ* is the viscous coefficient of air, *t* is the thickness of the MPNM, *σ* is the perforation rate, *d* is the perforation diameter, *k_m_* is the perforation constant, *δ_s_* is the perforated acoustic impedance end correction coefficient for perforated holes with a non-circular edge geometry, *δ_H_* is the non-adhesive acoustic impedance end correction factor, and t^*^ is the dimensionless plate thickness.

Secondly, from the microscopic point of view, the MPNM was made of interwoven nanofibers with a large number of pores distributed inside. The viscous effect generated by the friction between the air inside the fiber material and the rigid medium, as well as the thermal effect between the air inside the fiber material and the fiber material, can be calculated by the JCA equivalent fluid model, and the corresponding equations are as follows [29,30]:

(15) $$\rho (\omega )=\rho \left[1+\frac{1}{j2\pi }(\frac{p}{\rho f})\sqrt{1+j\pi (\frac{p}{\rho f})}\right]$$

(16) $$\begin{array}{l} K(\omega )=\gamma P_{0}\left\{\gamma -(\gamma -1)/{\left[1+\frac{1}{j8\pi N_{pr}{\left(\frac{p}{\rho f}\right)}^{-1}\sqrt{1+4j\pi (\frac{p}{\rho f})N_{pr}}}\right]}^{-1}\right\} \end{array}$$

where *ρ*(*ω*) is the effective density, *K*(*ω*) is the dynamic bulk modulus, *γ* is the heat capacity ratio of the air, *P*_0_ is the equilibrium pressure of the air, *N_pr_* is the Prandtl number, and *p* is the flow resistivity. The propagation constant *k_n_* of the acoustic wave and the acoustic impedance *Z_n_* of the MPNM are, respectively,

(17) $$k_{n}=2\pi f\sqrt{\frac{\rho (\omega )}{K(\omega )}}$$

(18) $$Z_{n}=\sqrt{\rho (\omega )K(\omega )}$$

Considering the influence of the microperforated structure, the viscous effect of the gas in the fiber material, and the thermal conduction effect between the gas and the medium on the sound absorption performance, the acoustic impedance rate Z*_PVB_* of the MPNM will become the parallel value of the two:

(19) $$Z_{PVB}={\left(\frac{1}{Z_{m}}+\frac{1}{Z_{n}}\right)}^{-1}$$

### 4.2. Transfer Matrix of the NFF

The transfer matrix of the NFF in the composite structure is

(20) $${\left[T\right]}_{PET}=\left[\begin{array}{cc} \cos(k_{a}D) & jZ_{PET}\sin(k_{a}D)\\ \frac{j}{Z_{PET}}\sin(k_{a}D) & \cos(k_{a}D) \end{array}\right]$$

where *k_a_* is the propagation constant of the acoustic wave, *D* is the thickness of the NFF, and *Z_PET_* is the acoustic impedance of the NFF. *k_a_* and *Z_PET_* can also be calculated by the theoretical model of the JCA through Equations (15)–(18).

### 4.3. Transfer Matrix of the MPNM-NFF

The transfer matrix of the MPNM-NFF is

(21) $$\left[T]=[T_{PVB}][T_{PET}\right]$$

Let *p_i_* and *v_i_* be the acoustic pressure and particle vibration velocity at the incident surface, and *p_i_*_+1_ and *v_i_*_+1_ be the acoustic pressure and particle vibration velocity at the reflecting surface; then, the relationship between the state variables on the leftmost MPNM and the variables on the rigid wall at the right end is

(22) $$\left[\begin{array}{l} p_{i}\\ v_{i} \end{array}\right]=\left[\begin{array}{cc} T_{11} & T_{12}\\ T_{21} & T_{22} \end{array}\right]\left[\begin{array}{l} p_{i+1}\\ v_{i+1} \end{array}\right]$$

Since the end of the composite structure was a rigid wall, the particle vibration velocity was zero. Form this boundary condition and the combination with the matrix transfer relationship between the acoustic pressure and the particle vibration velocity can be obtained as the acoustic impedance of the surface of the multilayer fiber membrane *Z* as follows:

(23) $$Z=\frac{T_{11}}{T_{21}}$$

Therefore, the SAC of the MPNM-NFF is

(24) α=4Re(Zρ0c0)(1+Re(Zρ0c0))2+Im(Zρ0c0)2

### 4.4. Comparison of Model-Based Predictions and Measured Values

**Figure 7:**
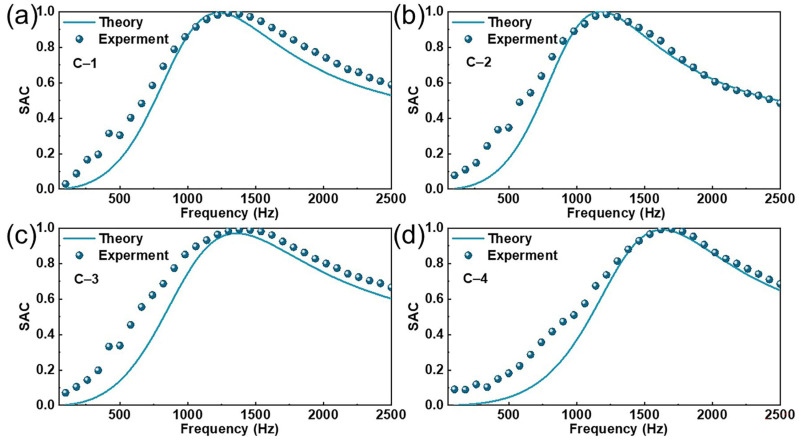
Comparison of the theoretical and measured values: (**a**–**d**) represent the SAC curves for the MPNM-NFF with different structural parameters.

To validate the accuracy of the model calculations, a numerical model of the SAC was created using MATLAB R2022b software for composite materials with different structural parameters, and the theoretical values were compared with the measured values. The results are presented in Figure 7, and the structural parameters of the different composite materials are listed in Table 2. As shown in Figure 7, the absorption model was able to predict the SAC of the MPNM-NFF within the range of 100–2500 Hz with good accuracy. The overall error was small, with the resonance frequency, peak absorption values, and the trend of the curves showing high overlap, indicating that the sound absorption mechanism had been comprehensively analyzed. The predictive performance of the model was quantitatively evaluated using the coefficient of determination (R^2^), Mean Absolute Error (MAE), and Root Mean Square Error (RMSE), as shown in Figure S1. The results indicated that the theoretical model exhibited high predictive accuracy, with R^2^ values for all the theoretical predictions and experimental data exceeding 0.95, demonstrating that the model effectively captured the trends in the SAC. Additionally, all MAE values were less than 0.1, and RMSE values were below 0.15, further confirming the robustness of the model. However, compared to the measured values, the theoretically calculated sound absorption bandwidth was slightly narrower, especially before the resonance frequency, where the measured values were consistently higher than the theoretical values. This discrepancy was primarily due to the irregularities of the nanofiber itself, which led to certain deviations in the improved theoretical model’s predictions. Nevertheless, this also served to confirm the superior sound absorption performance of the MPNM-NFF.

### 4.5. Optimization of Sound Absorption in the MPNM-NFF

The acoustic performance of the MPNM-NFF was closely linked to its structural parameters. By adjusting key factors such as the perforation diameter, perforation rate, thickness of the MPNM, and thickness of the nonwoven felt fabric the acoustic absorption properties can be finely tuned to achieve specific noise reduction goals. To further optimize the absorption performance across different frequency ranges, a particle swarm optimization algorithm was employed to refine the acoustic structure. The optimization aimed at maximizing the average SAC within low-frequency (100–1000 Hz), mid-frequency (1000–2500 Hz), and full-frequency (100–2500 Hz) bands, with the ultimate objective of identifying the most effective structural parameters for each range. Among them, the parameter ranges of the optimized structure were

(25) $$\left\{\begin{array}{l} 0.4\;\mathrm{mm}\leq d\leq 1\;\mathrm{mm}\\ 0.5\%\leq \sigma \leq 2\%\\ 0.5\;\mathrm{mm}\leq t\leq 1.5\;\mathrm{mm}\\ 5\;\mathrm{mm}\leq D\leq 20\;\mathrm{mm} \end{array}\right.$$

where *d*, *p*, *t*, and *D* represent the pore diameter, perforation rate, MPNM thickness, and NFF thickness, respectively. It is important to note that the ranges of these parameters are defined based on the multi-dimensional constraints derived from practical engineering applications, which include factors such as the fabrication feasibility, cost-effectiveness, and spatial compatibility.

**Figure 8:**
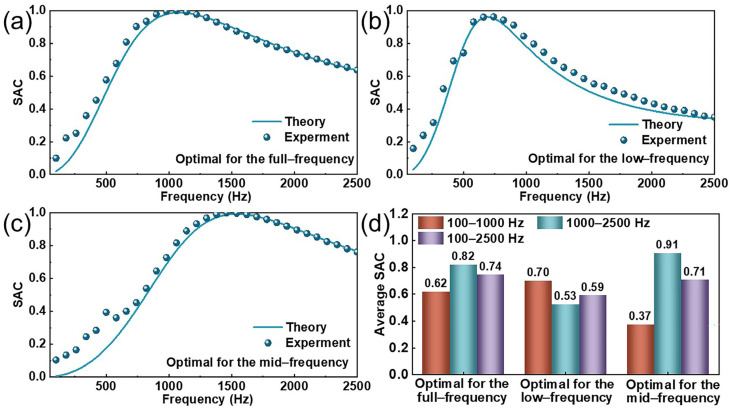
Simulated and measured values of the optimized MPNM-NFF: (**a**–**c**) SAC curves and (**d**) average SAC.

Table 3 presents the optimization results for the structural parameters, while Figure 8 illustrates the optimal SAC curves and corresponding average SAC for the MPNM-NFF across low-, mid-, and full-frequency ranges. As shown in Figure 8, by adjusting the perforation diameter, perforation rate, thickness of the MPNM, and thickness of the NFF, different sound absorption effects can be achieved. Furthermore, the algorithm-driven optimization of the structural parameters significantly enhanced the sound absorption performance of the composite structure. The optimal average sound absorption coefficient of the low-frequency range can be up to 0.70, the optimal average coefficient of the mid-frequency range can be up to 0.91, and the optimal average coefficient of the full-frequency range can be up to 0.74. Additionally, Figure S2 presents the fitting analysis results between the experimental values and the predicted values. As shown, both the R^2^ values for the theoretical predictions and experimental data exceeded 0.95, and both the MAE and RMSE were below 0.1, further confirming the high predictive accuracy and robustness of the model. In the practical application, the calculation procedure of the optimization algorithm can be used to guide the design process. By considering the noise frequency range and the challenges associated with sample preparation, the frequency range and structural parameters were adjusted to achieve the optimal parameter configuration for specific noise bands, thereby providing more precise and effective solutions for noise control engineering.

**Figure 9:**
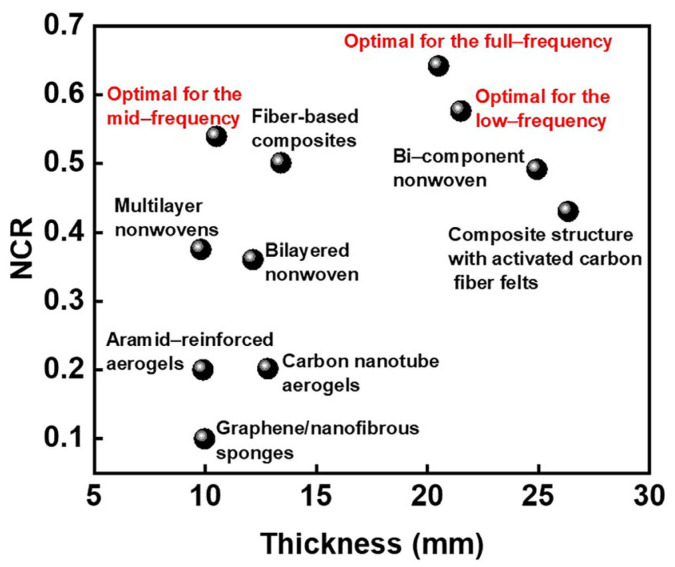
The NRC of the MPNM-NFF and other sound-absorbing materials [31,32,33,34,35,36,37,38].

Figure 9 shows that the MPNM-NFF fabricated in this study exhibits a significant advantage in NRC values compared to other sound-absorbing materials reported in the literature [31,32,33,34,35,36,37,38]. Notably, at the same thickness, the NRC of the MPNM-NFF was substantially higher than that of other materials, directly reflecting its superior noise reduction performance. Compared to existing products, the MPNM-NFF offered an enhanced noise reduction capability while maintaining lightweight and flexible properties. These features made it more advantageous in practical production and application, facilitating its broader adoption.

## 5. Conclusions

To achieve efficient sound absorption with lightweight, flexible fiber materials, this study developed a composite of MPNM-NFF. This composite material fully exploited the resonance effect of the micro-perforated nanofiber membrane and the viscous effect of the fibrous porous material, forming a fiber-based composite sound-absorbing structure with multiple absorption mechanisms. Its lightweight, thin, and flexible characteristics offered a promising approach for better control of mid-to-low-frequency broadband noise within limited spaces. Additionally, by analyzing the sound absorption mechanism of the composite material, a theoretical model for calculating the SAC was established, which effectively predicted and analyzed the sound absorption performance of the MPNM-NFF in the 100–2500 Hz range. The resonance frequency, absorption peak, and the trend of the absorption curve showed high agreement, providing a solid theoretical foundation for structural design. Furthermore, through a combination of theoretical analysis and experimental validation, the study investigated the effects of the lamination sequence, bonding area, perforation parameters, thickness of the MPNM, and thickness of the NFF on sound absorption performance. Finally, an optimized structural parameter combination was determined through algorithmic optimization to achieve superior noise reduction performance across different frequency bands, providing targeted absorption for various noise levels and offering strong support for practical applications.

## Figures and Tables

**Table 1:** Structural parameters of test samples.

Sample	MPNM Thickness/mm	NFF Thickness/mm	Perforation Rate/%	Perforation Diameter/mm	Cavity Depth/mm
MPNM	0.5	-	1	0.8	-
MPNM-AIR	0.5	-	1	0.8	10
NFF	-	10	-	-	-
MPNM-NFF	0.5	10	1	0.8	-
NFF-MPNM	0.5	10	1	0.8	-

**Table 2:** Structural parameters of the test samples.

Sample	Perforation Rate/%	Perforation Diameter/mm	MPNM Thickness/mm	NFF Thickness/mm
C-1	1	0.6	0.5	10
C-2	1	0.8	0.5	10
C-3	1.5	0.8	0.5	10
C-4	1	0.8	0.5	5

**Table 3:** Optimization results for the structural parameters of the MPNM-NFF.

Structure	Perforation Rate/%	Perforation Diameter/mm	MPNM Thickness/mm	NFF Thickness/mm
Optimal for the full-frequency range	2	0.4	0.5	20
Optimal for the low-frequency range	1.8	0.4	1.5	20
Optimal for the mid-frequency range	2	0.4	0.5	10

## Data Availability

The original contributions presented in this study are included in the article. Further inquiries can be directed to the corresponding author(s).

## Supplementary Materials

The following supporting information can be downloaded at https://www.mdpi.com/article/10.3390/polym17070874/s1: Figure S1: Fitting analysis: (a–d) represent the relationship between the theoretical and measured SAC for the MPNM-NFF with different structural parameters; Figure S2: Fitting analysis: (a–c) represent the relationship between the theoretical and measured SAC for the MPNM-NFF with different structural parameters.
