# Supplementary material for: Simulation, Measurement, and Optimization of Sound Absorption in Nanofiber Membrane Composite with a Nonwoven Material
Source: Polymers (Basel). 2025 Mar 25;17(7):874. doi: 10.3390/polym17070874 (PMC11991465; doi:10.3390/polym17070874)
Supplement: Supplementary file 1 [file polymers-17-00874-s001.zip › polymers-3532444-supplementary.pdf]

Simulation, measurement and optimization of sound absorption in nanofiber membrane composite nonwoven material

Xiaofei Shao and Xiong Yan *
Key Laboratory of Textile Science and Technology, Ministry of Education, College of Textiles, Donghua University, Shanghai 201620, China; shaoxiaofei@mail.dhu.edu.cn
* Correspondence: yaxi@dhu.edu.cn

Supplementary Information

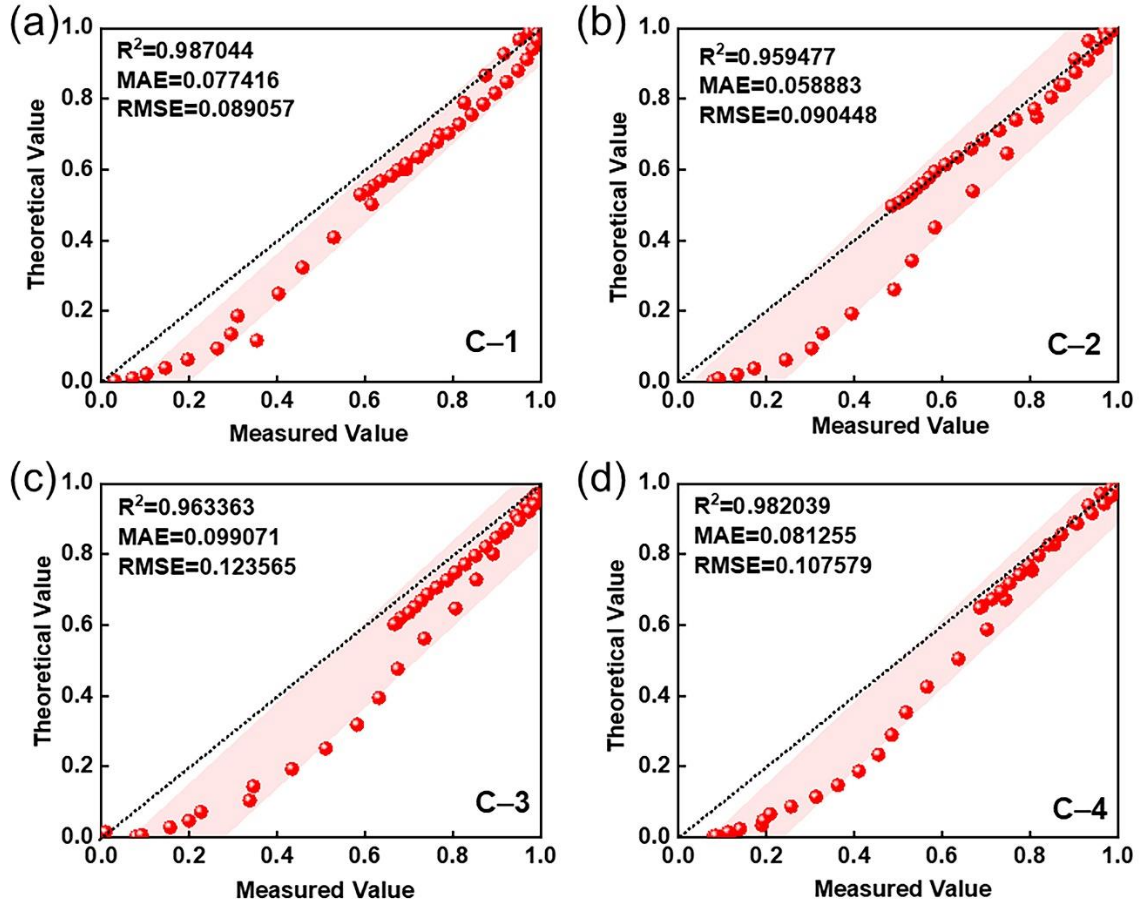

Figure S1: Fitting analysis: (**a**)-(**d**) represent the relationship between the theoretical and measured SAC for the MPNM-NFF with different structural parameters.

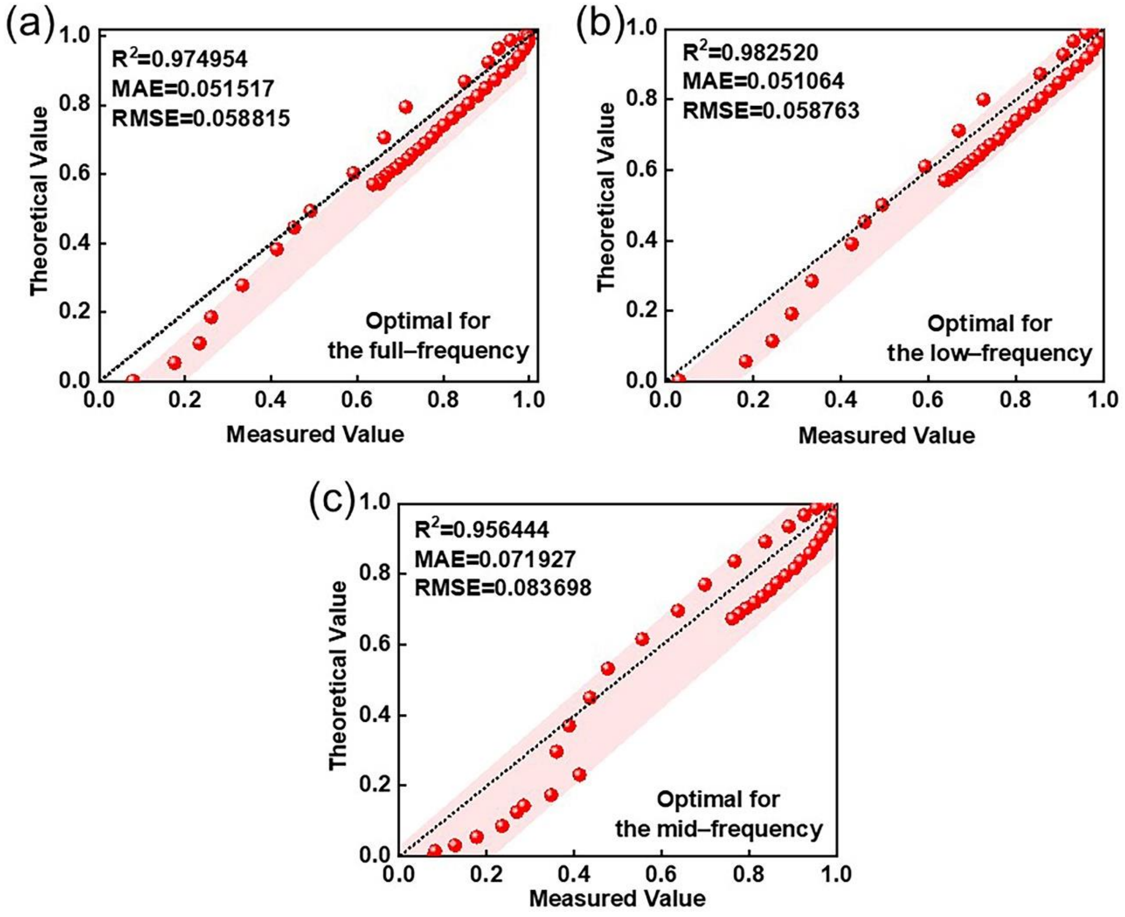

Figure S2: Fitting analysis: (**a**)-(**c**) represent the relationship between the theoretical and measured SAC for the MPNM-NFF with different structural parameters.
